# Metabolomic identification of biochemical changes induced by fluoxetine and imipramine in a chronic mild stress mouse model of depression

**DOI:** 10.1038/srep08890

**Published:** 2015-03-09

**Authors:** Jing Zhao, Yang-Hee Jung, Choon-Gon Jang, Kwang-Hoon Chun, Sung Won Kwon, Jeongmi Lee

**Affiliations:** 1 School of Pharmacy, Sungkyunkwan University, Suwon 440-746, Republic of Korea; 2 Gachon Institute of Pharmaceutical Sciences,College of Pharmacy, Gachon University, Incheon 406-799, Republic of Korea; 3 College of Pharmacy, Seoul National University, Seoul 151-742, Republic of Korea

## Abstract

Metabolomics was applied to a C57BL/6N mouse model of chronic unpredictable mild stress (CMS). Such mice were treated with two antidepressants from different categories: fluoxetine and imipramine. Metabolic profiling of the hippocampus was performed using gas chromatography-mass spectrometry analysis on samples prepared under optimized conditions, followed by principal component analysis, partial least squares-discriminant analysis, and pair-wise orthogonal projections to latent structures discriminant analyses. Body weight measurement and behavior tests including an open field test and the forced swimming test were completed with the mice as a measure of the phenotypes of depression and antidepressive effects. As a result, 23 metabolites that had been differentially expressed among the control, CMS, and antidepressant-treated groups demonstrated that amino acid metabolism, energy metabolism, adenosine receptors, and neurotransmitters are commonly perturbed by drug treatment. Potential predictive markers for treatment effect were identified: myo-inositol for fluoxetine and lysine and oleic acid for imipramine. Collectively, the current study provides insights into the molecular mechanisms of the antidepressant effects of two widely used medications.

Depression is a prevalent complex psychiatric disorder that can lead to emotional and physical problems, including loss of interest or pleasure, feelings of guilt or low self-worth, and disturbed sleep or appetite^1^. Because it is a major cause of disability, suicide, and physical disorders^2^, patients are prescribed antidepressants, which are psychiatric medications used to alleviate mood and behavioral symptoms. Most of the widely prescribed classes of antidepressants are selective serotonin reuptake inhibitors (SSRIs) such as fluoxetine, paroxetine, and sertraline^3^, serotonin–norepinephrine reuptake inhibitors (SNRIs) such as venlafaxine, desvenlafaxine, and duloxetine^4^,^5^,^6^, and tricyclic antidepressants (TCAs) such as imipramine, clomipramine, and desipramine^7^,^8^,^9^,^10^.

The chronic mild stress (CMS) model is a valuable animal model of depression, given that the model animals mimic several human symptoms of depression^11^. In this model, animals are exposed sequentially to a variety of unpredicted, mild stressors for several weeks. Following exposure to a series of stressors, many behavioral and biochemical changes subsequently occur, which are reversible with antidepressant treatments^12^. Therefore, the CMS model of depression is considered to be suitable for investigating the pathophysiology of depression and antidepressant effects of diverse drugs^13^,^14^.

Metabolomics investigates the metabolic response of living systems to any stimuli by measuring variations in the metabolic profiles of the biofluids and tissues of an organism. It has been increasingly used as a versatile tool for discovery of molecular biomarkers in many areas including diagnosis or prognosis of clinical diseases and investigation of potential mechanisms of diseases and drugs^15^,^16^. Metabolomic studies are promising tools for the identification of metabolite alterations on stress and drug administration^16^,^17^,^18^,^19^. A number of metabolomic studies have been reported on various types of samples from animal models of CMS. Biological fluid samples such as urine^20^ and plasma^21^ have been preferably used in metabolomics studies, including studies of depression, because these samples are obtained in a minimally invasive manner and therefore are clinically practical. On the other hand, tissues also serve as useful samples in metabolomics despite the invasive sampling required, in that they can provide unique, localized, and more relevant metabolic information from the sample of interest. Therefore, metabolomics studies have also been done with the brains of CMS model mice^3^,^22^. Previously, molecular alterations induced by traditional Chinese prescriptions for mental disorders have been examined in the urine or plasma of CMS model rats^18^,^23^ and metabolic profiling has been performed in the hippocampus of DBA/2 mice chronically treated with paroxetine, an SSRI class drug^24^.

Efficacy was compared between two popular antidepressants of different classes, fluoxetine (SSRI class) and imipramine (TCA class) in the treatment of inpatient depression, revealing that fluoxetine was as effective as imipramine in terms of response and remission rates^25^. Nonetheless, no comparative studies have yet provided insight into antidepressant mechanisms using a relevant model system. The hippocampus, a brain region involved in memory and mood control, is the area most associated with depression, as reported in a large number of preclinical and clinical studies^26^,^27^,^28^. For example, the hippocampus was structurally and functionally affected in a chronic stress model of depression, and antidepressants are known to reverse the morphological changes induced by chronic stress^29^,^30^. Chronic fluoxetine treatment increased hippocampal neurogenesis in adult rats^28^.

The discovery of biomarkers that indicate antidepressant treatment efficiency at an early stage is in great demand; thus, clinical specimens such as plasma and urine, which require only minimally invasive sampling, could be very informative and useful. However, the central goal of our metabolomics study was to investigate metabolic changes in the hippocampus, the brain region most associated with depression, as well as behavioral responses to depression and antidepressant treatment in order to provide detailed mechanistic insight into depression and the antidepressant effects of fluoxetine and imipramine. We hypothesized that treatment of CMS-treated mice with antidepressants of different classes would alter metabolic profiles of the hippocampus and/or stress-related behaviors in different manners. To test this hypothesis, we established four groups of C57BL/6N strain mice, which is typically used for depression-related behavior tests^31^ and the chronic stress mouse model^32^,^33^. The groups comprised a control (unstressed) group, CMS (stressed) group, and CMS groups treated with either fluoxetine or imipramine. In parallel with body weight measurement and behavior tests including the open field test (OFT) and forced swimming test (FST), we performed a gas chromatography-mass spectrometry (GC-MS)-based metabolomics study in a CMS mouse model. The entire experimental design is displayed in Fig. 1. To our knowledge, this is the first systematic analysis comparing metabolomic changes after sub-chronic treatment with fluoxetine and imipramine.

## Results

### Behavior tests and body weight measurement

**Figure 1:**
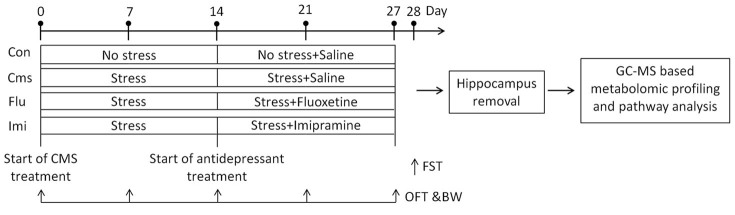
Experimental design for the present study.

**Figure 2:**
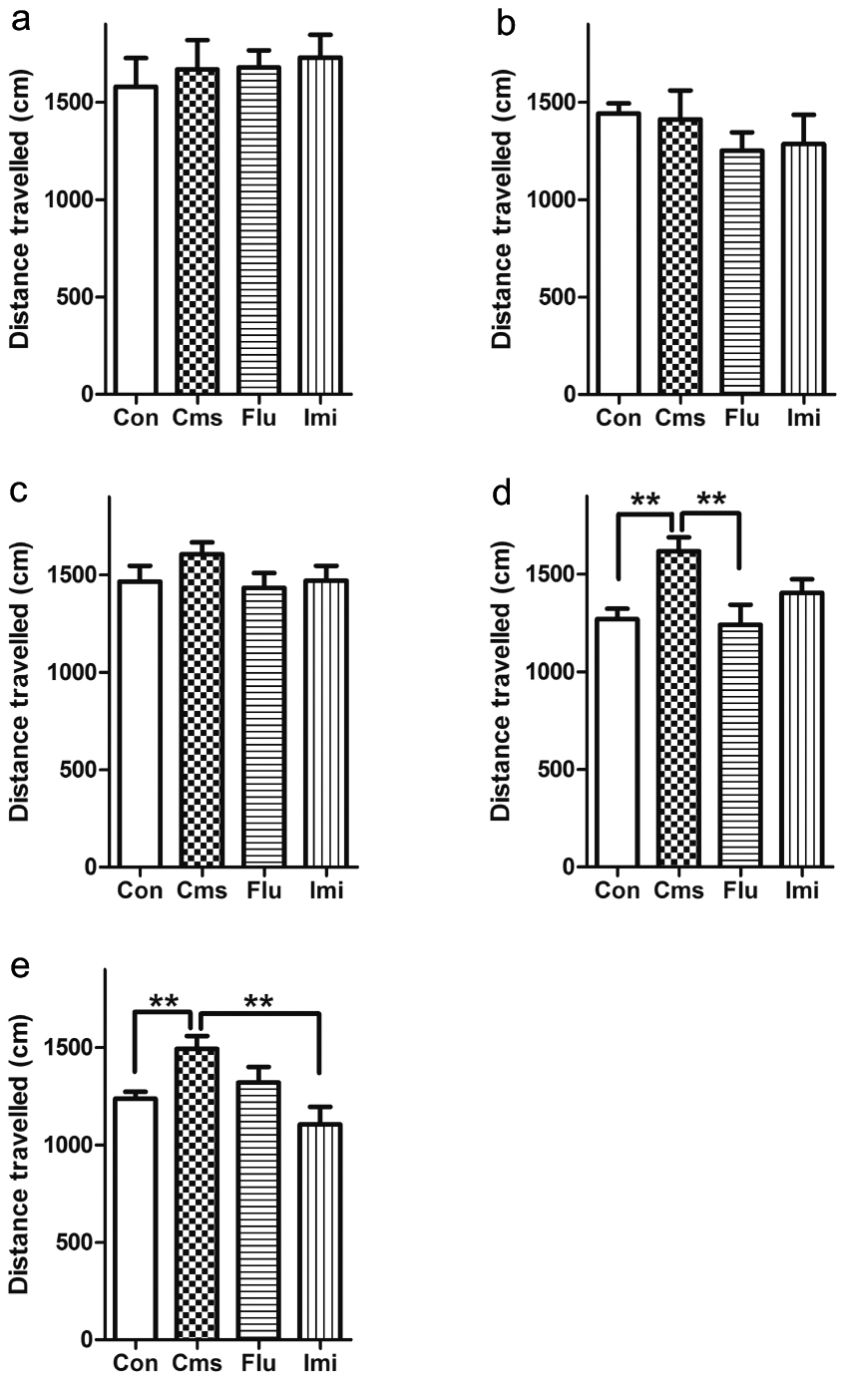
Measurement of total-travelled distance in the open field test at day 0 (a), 7 (b), 14 (c), 21 (d), and 27 (e) of the CMS procedure. ** indicates *p* < 0.01. (Con, control group treated with saline; Cms, CMS group treated with saline; Flu, fluoxetine-treated group; Imi, imipramine-treated group).

Locomotor activity was measured weekly during the stress period (day 0, 7, 14, 21, and 27) as total-travelled distance in the OFT to evaluate the stress-related status of the mouse model (Figs. 1 and 2). Mean total-travelled distance of control and drug-treated groups had a tendency to decrease over time during the stress period. However, the total distance of the stressed group with no drug treatment (Cms) generally remained unchanged. As a result, the total-travelled distance of the Cms group was significantly higher than that of the control group at day 21 and day 27 (*p* < 0.01). At day 21, which was one week after drug treatment, the total distance of the fluoxetine-treated (Flu) group was significantly decreased compared to the Cms group (*p* < 0.01), while a noticeable, but insignificant difference was observed between the Cms and imipramine-treated (Imi) groups (*p* = 0.0520) (Fig. 2d). On the last day of the stress period (day 27), the total-travelled distance of the Imi group was reduced to a level close to the control group. In the meantime, travel distance was lower in the Flu than the Cms group, but this difference was not statistically significant at day 27.

**Figure 3:**
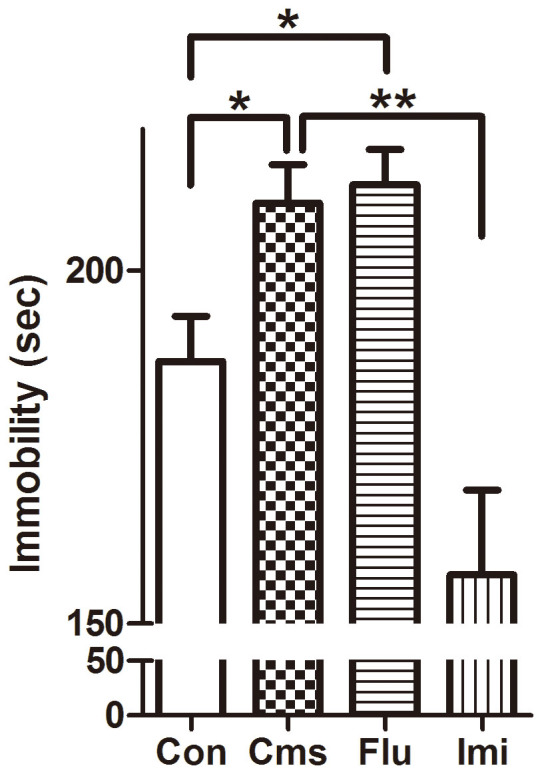
Measurement of immobility (floating) time in the forced swimming test at the end of CMS procedure. * indicates *p* < 0.05 and ** indicates *p* < 0.01.

The FST is a highly reliable test and has strong validity for indicating depressive-like behavioral status and antidepressant effects in CMS animal models^34^. In the FST, immobility (floating) time was measured as an estimate of phenotypes of depression. In Fig. 3, floating time was significantly elevated in the Cms group compared to control (*p* < 0.05) and sub-chronic imipramine treatment significantly reduced immobility (*p* < 0.01). However, the Flu group spent a similar amount of time floating compared to the Cms group.

Changes in body weight were measured during the stress period (Supplementary Fig. S1). CMS caused significant weight loss in Cms group mice (day 14, 21, and 27; *p* < 0.001). The Flu and Imi group mice also experienced significant weight loss after two weeks of stress before treatment (day 14; *p* < 0.05). However, after the Flu and Imi groups started to receive antidepressants, their body weight returned to normal (control) levels in both of those groups.

### Metabolic profiling of the hippocampus by GC-MS

#### Optimization of sample preparation for GC-MS analysis

In order to increase the efficiency of sample preparation, several parameters were optimized including type of extraction solvent and derivatization conditions. Among the tested extraction solvents (methanol, ethanol, 10 mM phosphate buffer, and mixture of chloroform-methanol-water (2:5:2)), the chloroform-methanol-water mixture yielded the largest number of peaks (data not shown) and therefore it was selected as the extraction solvent.

For GC-MS-based metabolic profiling of hippocampus tissues, the sample extracts underwent chemical derivatization using the most commonly applied reactions, methoximation followed by trimethylsilylation^35^. Volumes of 30 mg mL^−1^ methoxyamine hydrochloride (50, 100, 300 μL) and BSTFA (50, 100, 300, 500 μL), and reaction temperature for trimethylsilylation (25, 37, 70°C) were systematically optimized to provide the largest number of peaks (data not shown). As a result, optimized derivatization conditions were 50 μL of 30 mg mL^−1^ methoxyamine hydrochloride solution in pyridine for 2 h at room temperature, followed by a reaction with 500 μL of BSTFA for 4 h at 37°C. These conditions were used for further metabolic profiling.

#### Metabolic profiles analyzed by GC-MS

To minimize any metabolic changes caused by environmental factors, inbred mice with homogeneous genetic backgrounds (C57BL/6N mice) were used for experiments under well-controlled conditions. Typical total ion chromatograms (TICs) of hippocampus samples from the four groups of mice are shown in Supplementary Fig. S2. Visual inspection of these spectra revealed differences in TIC profiles among the four groups, indicating that the endogenous metabolite levels were perturbed by CMS and antidepressants. Based on mass alignment with a mass spectral database and comparison to authentic standards and data from the literature, 38 metabolites were identified in this study, including amino acids, fatty acids, sugars, and organic acids (Supplementary Table S1).

#### Multivariate statistical analysis of the metabolomics data

**Figure 4:**
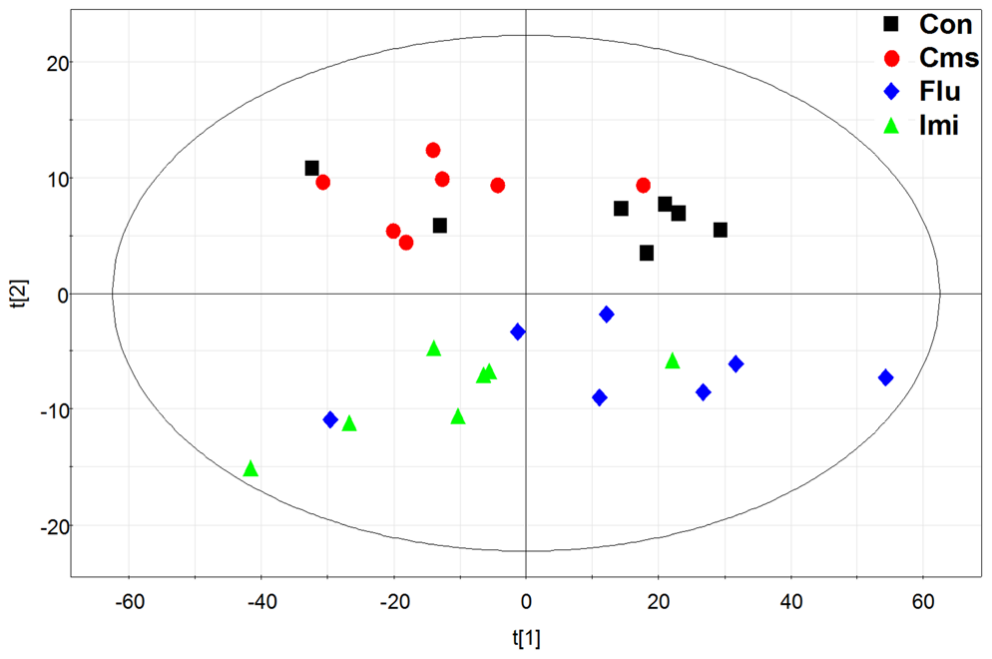
PCA score plot derived from the GC-MS analysis of hippocampi from control, Cms, Flu, and Imi groups. (black squares, control; red circles, Cms; blue diamonds, Flu; green triangles, Imi).

A PCA was used to visualize general clustering, trend, or outliers among the observations. Score plot and model analysis including Hotelling's T2 plot revealed no outliers. As seen in Supplementary Fig. S3, quality control samples were tightly clustered, which suggests that the quality of data was acceptable^36^. In the PCA score plot, the two drug-treated groups were separated from the Con and Cms groups, but no discernible clustering was observed between the Con and Cms groups or between the Flu and Imi groups (Fig. 4).

**Figure 5:**
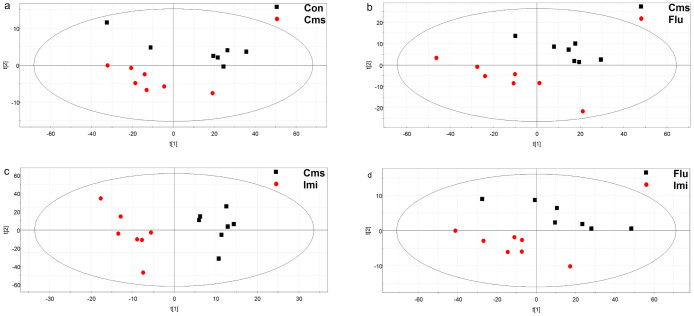
PLS-DA score plots for pair-wise comparisons between control and Cms (a), Cms and Flu (b), Cms and Imi (c), and Flu and Imi groups (d).

A PLS-DA was applied to better understand the different metabolic patterns and to detect potential biomarkers showing prominent concentration changes in the models. Quality of the resulting discriminant models is summarized in Table 1. The key model parameters, R^2^ and Q^2^ in pair-wise groups were larger than 0.5, suggesting that all models were robust and had good fitness and prediction. The control and Cms groups were clearly distinguished in the PLS-DA plot (Fig. 5a). PLS-DA score plots (Figs. 5b, 5c) showed that the drug-treated groups had distinctive metabolic profiles from the Cms group and clear separation was also observed between the Flu and Imi groups (Fig. 5d).

#### Potential marker metabolites for depression and antidepressant effects

The OPLS-DA S-plot and variable importance for projection (VIP) statistics were used for selecting significant variables responsible for group separation^37^,^38^. Variables were pre-selected as candidates when their VIP values were larger than 1.0. Then, among these, variables with |Corr (*t*, X)| > 0.58 in S-plot (Supplementary Fig. S4) were then selected as variables that were most correlated with the OPLS-DA discriminant scores^3^ in order to decrease the risk of false positives in the selection of potential biomarkers^39^. Applying such an approach, it was possible to identify metabolites important for discriminating depression and drug types. The identified potential markers are listed in Table 2. Except for the four compounds for which peak identification was unsuccessful, a total of 19 marker compounds were discovered.

In the evaluation of the Cms group against control, seven metabolites were significantly altered: urea, phosphoric acid, glutamine, and cholesterol were up-regulated and N-carboxy-glycine, hexadecanoic acid, and octadecanoic acid were down-regulated in Cms group. Treatment of stressed mice with antidepressants induced more obvious metabolic perturbations (Supplementary Fig. S2 and Table 2). Levels of 12 hippocampus metabolites of known identity differed between the Flu and Cms groups. Flu versus Cms mice exhibited higher levels of two fatty acids, hexadecanoic and octadecanoic acids, which had been down-regulated by depression. Besides these fatty acids, levels of malic acid, leucine, and glycine were also increased, while levels of myo-inositol, adenosine, N-acetyl-aspartic acid (NAA), glutamic acid, and creatinine were lower in the Flu compared to the Cms group.

Imipramine treatment induced more noticeable metabolic changes than fluoxetine. Fourteen metabolites were expressed at significantly different levels. Hexadecanoic acid, octadecanoic acid, leucine, butanoic acid, and glycine were up-regulated while adenosine, NAA, and glutamic acid were down-regulated. Perturbation in the levels of these eight metabolites was similarly found in the comparison between the Flu and Cms groups. In the Imi group, however, five more metabolites were newly detected as significant metabolites. Levels of valine, aspartic acid, lysine, and oleic acid were significantly increased and the level of urea was reduced compared to the Cms group. These results strongly suggest that CMS and antidepressants disturb these metabolic pathways in mice in different ways.

## Discussion

The significantly increased locomotor activity of Cms mice compared to control mice from day 21 in the OFT indicated hyperactivity induced by chronic stress in the CMS model^18^. Intriguingly, degrees of reduction in total-travelled distance by imipramine and fluoxetine were fairly different; at day 21, only fluoxetine significantly reduced total-travelled distance, whereas only imipramine significantly reduced this variable at day 27. These observations indicate that psychomotor activity was disturbed by CMS and that the two types of antidepressants reversed the effect of CMS to different degrees in a time-dependent manner.

In the FST, immobility was significantly reduced in the Imi group, implying that imipramine reversed the depression-like symptoms of stressed mice. In constrast, fluoxetine failed to induce noticeable change in floating time. Possible explanations for the apparent lack of fluoxetine effect in the FST are that C57BL/6 mice were less responsive to fluoxetine than other mouse strains in this model, similar to Lucki et al.'s report^40^, or that the duration of fluoxetine treatment was not long enough considering that SSRIs generally exhibit slow onset of action in clinical patients^41^. Other minor factors also might have affected response results, including sub-strain differences and administration-related factors (e.g., administration method, dose, and period), or differences in laboratory protocols, equipment, and mouse handling^31^,^32^. Because the current study was designed to compare two representative antidepressants from different classes, the same sub-chronic administration regimen was applied to both Flu and Imi groups during the last half of the stress period. The FST alone likely was not enough to confirm the antidepressant effect in the present model. A more comprehensive study is currently under design that will investigate metabolic and behavioral changes in hippocampus as well as blood and urine upon acute and chronic administration of fluoxetine using a CMS mouse model of C57BL/6N and another strain such as BALB/c.

Significant body weight loss in the Cms group from day 14 through day 27 was probably due to loss of appetite as similarly found in human patients with depression; weight loss is typically indicative of depression in the mouse model. The return to normal body weight with fluoxetine and imipramine treatment partially supports their antidepressant effects on the stressed mice. Collectively, these findings confirmed that a depressive-like status had been developed in the CMS model and that the two drugs had antidepressant effects on the stressed mice, but to different degrees.

**Figure 6:**
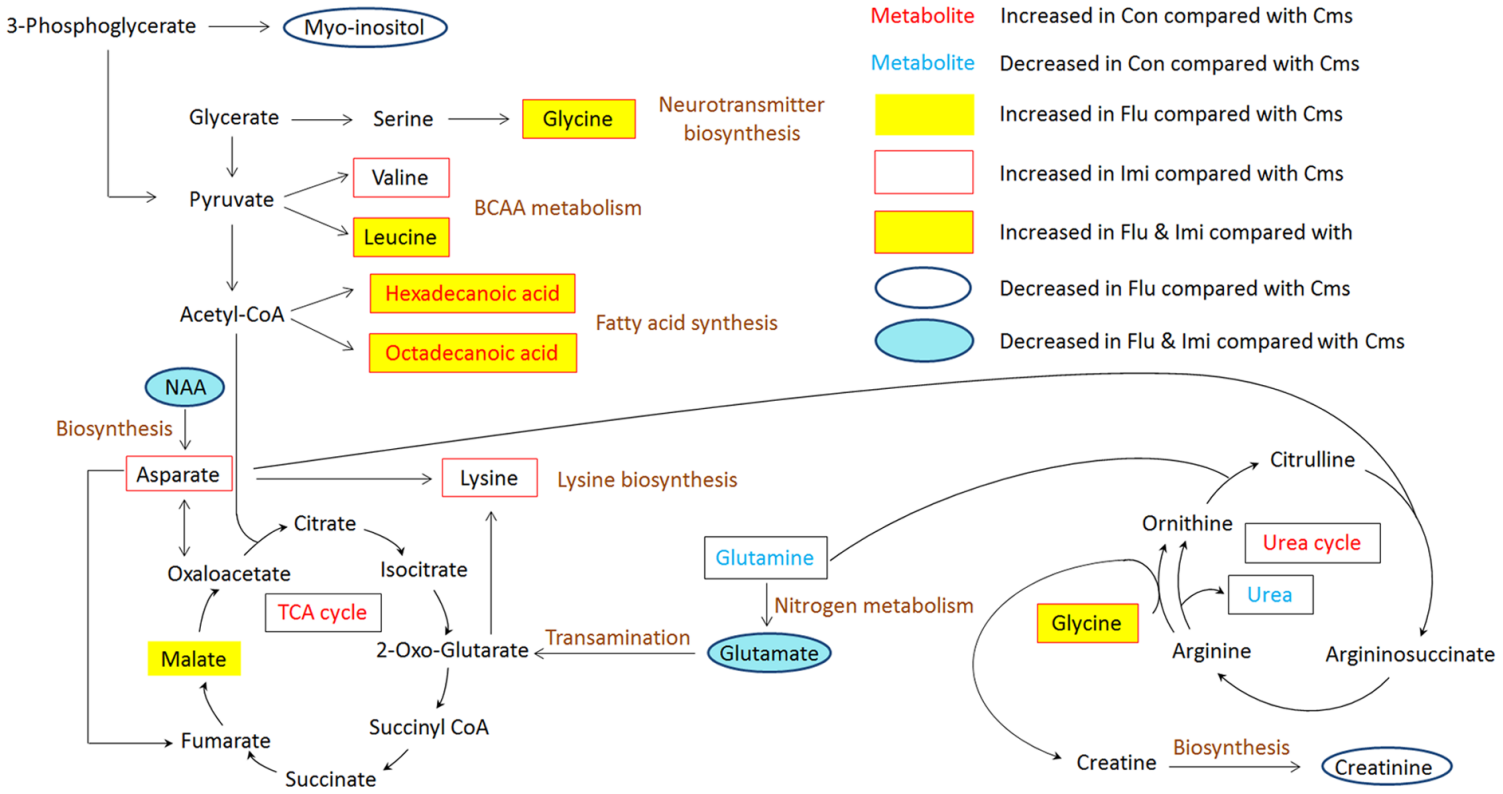
Metabolic pathways affected by CMS and/or sub-chronic treatment with fluoxetine and imipramine.

Our GC-MS-based metabolomics investigation of the hippocampus revealed that metabolic perturbation occurred from stress and sub-chronic administration of antidepressants. Twenty-three metabolites were found related to depression and/or antidepressant effects of fluoxetine and imipramine and a summary figure for the affected metabolites in the related metabolic pathways is displayed in Fig. 6.

When comparing control and Cms groups, relative levels of several metabolites were altered, contributing to different metabolic profiles. All the identified metabolites responsible for this difference except for N-carboxy-glycine were previously detected in plasma profiles of CMS model rats despite the different sample types^19^, likely because of the technical characteristics of GC-MS used for both studies. In comparison to control, glutamine was up-regulated in Cms mice. Although differentially expressed glutamine levels were reported depending on the model species and brain region^18^,^22^,^42^, disturbances in glutamine metabolism indicate that changes in glutamatergic neurotransmission are associated with depression^43^,^44^. In contrast to elevated glutamine levels, hexadecanoic acid and octadecanoic acid were significantly lower in the Cms group than the control group, while treatment with fluoxetine and imipramine significantly elevated the levels of these fatty acids compared to the Cms group. Decreased fatty acid levels in this study appear related to a deficiency in energy, i.e., fatigue, which is one of the most characteristic symptoms of depression.

Treatment of CMS mice with antidepressants induced significant alterations in biochemical profiles as well as in behaviors and body weight. Overall, our study results indicate that the two antidepressants exhibit therapeutic effects through the downstream pathways associated with amino acid metabolism and neurotransmission. Changes in amino acid metabolism were prominent among the affected metabolisms; seven out of 16 significant metabolites accountable for class discrimination were amino acids, including glycine, valine, leucine, glutamic acid, NAA, aspartic acid, and lysine.

Glutamic acid was the amino acid most significantly reduced by drug administration in comparison to the Cms group and its level was close to that of the control group (Supplementary Table S2). In contrast to the traditional monoamine hypothesis, recent studies have recognized the importance of the glutamatergic system associated with the pathophysiology of mood disorders, with glutamic acid playing a central role in the neuroplasticity hypothesis of mood disorders^45^. The two tested drugs in the current study apparently exerted antidepressant effects by modulating glutamate release in the mouse model.

NAA in the drug-treated mice returned to levels close to the control group, but it was still significantly lower than that of the Cms group. NAA is synthesized from aspartic acid and acetyl coenzyme A in neurons and its concentration is one of the highest of all free amino acids, indicating its important role in brain metabolism^46^. The current results imply that NAA was somehow up-regulated by depression. However, due to contradictory observations on NAA levels in CMS models depending on brain region and analytical method^3^,^47^,^48^, more studies of the CMS mouse model will be needed for accurate assessment of the effects of CMS and fluoxetine or imipramine on hippocampal NAA levels.

Glycine, an inhibitory neurotransmitter, was shown to have beneficial effects in the treatment of depression^49^. Related to these observations, we found that glycine levels were increased with fluoxetine and imipramine treatment compared to the Cms group. Levels of adenosine, which functions as a fine-tuning neuromodulator in neuronal communication^50^, were significantly disturbed by the antidepressants, indicating that the two drugs may share a common downstream pathway involving adenosine receptors^50^,^51^.

Two branched-chained amino acids (BCAAs), leucine and valine were also identified as markers. As compared to the Cms group, valine was up-regulated in the Imi group while leucine increased by 2-3-fold in both the Imi and Flu groups. Elevated levels of BCAAs were associated with lowered 5-hydroxytryptamine (5-HT) level because BCAAs can compete with tryptophan, a precursor of 5-HT, for transport across the blood-brain barrier, and therefore it was suggested that BCAAs can reduce central fatigue^52^. BCAA metabolism is also directly related to energy metabolism.

Aspartic acid is an excitatory neurotransmitter as well as a precursor to oxaloacetic acid, which is an important intermediate in the tricarboxylic acid cycle (TCA). Aspartic acid was increased only in the Imi group in comparison to the Cms group, implying that the therapeutic effect of imipramine involves neurotransmitter and energy metabolism. Consequently, it is likely that fluoxetine and imipramine differentially altered energy metabolism and the production of amine neurotransmitters, both of which appear related to fatigue as a depressive symptom^24^. In addition to aspartic acid, lysine was also up-regulated by only imipramine treatment. Intriguingly, the “anti-stress” effect of lysine was reported in a rat model^53^ and lysine supplementation in lysine-deficient humans significantly alleviated anxiety^54^. Apparently the stronger stress-alleviating effects of imipramine than fluoxetine during the FST and OFT that we observed may be related to the increased lysine level in the Imi group. Besides hexadecanoic and octadecanoic acids, which were commonly identified as differential markers for both antidepressants, oleic acid was identified as a distinctive marker for the Imi group, which displayed an increased level of oleic acid. Related to this result, the preventive or treatment effect of oleic acid was indicated for depressive order in humans^55^.

Malic acid and myo-inositol were identified as potential markers differentiating the treatment effects between fluoxetine and imipramine. Up-regulation of malic acid and down-regulation of myo-inositol compared to the Cms group were observed only in the Flu group. It was previously reported that increased malic acid level is associated with symptom reduction in depression^56^. Myo-inositol, a key metabolic precursor to the phosphoinositide pathway, is associated with psychiatric disorders and has therapeutic effects on SSRI-sensitive disorders including depression and related anxiety disorders^57^. It was previously identified as a predictive marker for the chronic treatment effect of paroxetine in mice^58^. Myo-inositol and fluoxetine, but not imipramine, similarly decreased the function of serotonin-2A receptor through G_q_ proteins *in vitro*^51^. These findings suggest that fluoxetine is different from imipramine by exerting its therapeutic effects through the phosphoinositide pathway, which is involved in signaling in serotonergic neurotransmission and in glucose and glycogen metabolism.

In summary, distinction between the depressed and control groups was observed in behaviors and metabolic patterns in the hippocampus, especially involving amino acid metabolism and energy metabolism. Sub-chronic treatment with imipramine and fluoxetine exerted therapeutic effects in the depressed mice to varying degrees, as indicated by behavior tests. Differentially expressed metabolites between the drug-treated and Cms groups indicated that the metabolic pathways involving amino acid metabolism, energy metabolism, adenosine receptors, and neurotransmitters were commonly perturbed by the drug treatment. Metabolic disparity observed between Flu and Imi groups strongly suggest that the different antidepressant effects are due to their differential effects on downstream pathways. The phosphoinositide pathway involving myo-inositol is likely to be one of the downstream pathways affected by fluoxetine. Lysine and oleic acid may be predictive markers of the treatment effect of imipramine. Collectively, the current study provides insights into the molecular mechanisms of antidepressant effects of two common medications in the CMS model of depression.

## Methods

### Chemicals

Pyridine, chloroform, methoxylamine hydrochloride, N,O-bistrimethylsilyltrifluoroacetamide (BSTFA) with 1% trimethylchlorosilance (TMCS), alanine-*d*_4_, ribitol, and imipramine were of analytical grade and were purchased from Sigma-Aldrich (St. Louis, MO, USA). Fluoxetine was obtained from TCI (Tokyo, Japan). HPLC-grade methanol (MeOH) was purchased from Duksan (Ansan, Korea). Doubly-distilled water was obtained using a Milli-Q water purification system from Millipore (Bedford, MA, USA). A Gyrozen centrifuge (Incheon, Korea) was used for centrifugation.

### Animals

Eight-week-old male C57BL/6N mice were purchased from Koatech Co., Ltd (Seoul, Korea). Mice were maintained in a temperature and humidity controlled room (23 ± 1°C, 55 ± 5%) under a 12 h light/dark cycle (lights on at 07:00–19:00) with access to food and water ad libitum. All animal care procedures were conducted in accordance with the US National Institutes of Health (NIH) Guide for the Care and Use of Laboratory Animals and were approved by the Institutional Animal Care and Use Committee of Sungkyunkwan University. After one week of acclimatization, mice were grouped for experiments.

### Chronic mild stress procedure and drug administration

Mice were randomly divided into four groups (n = 7 per group): control group (Con), CMS model group (Cms), fluoxetine-treated group (Flu), and imipramine-treated group (Imi). CMS consisted of exposure to the following stressors in a random order for 27 days: tilt cage, confinement, soiled bedding, white noise, removal of nesting materials, paired housing, reversed light-dark cycle, and overnight illumination^59^. All groups except for the control group were exposed to CMS twice a day from day 1 to day 27. From day 15 to day 27, Flu and Imi mice received fluoxetine and imipramine, respectively, at a dose of 20 mg/kg by intraperitoneal injection, while Con and Cms mice received the same volume of vehicle (saline solution) once a day.

### Experimental design

The design of the whole experiment was displayed in Fig. 1. The body weight of each mouse in four groups (Con, Cms, Flu, and Imi) was measured weekly (day 0, 7, 14, 21, and 27 of CMS application). OFT was also performed weekly, while the FST was performed only at the last day (day 28) prior to sacrifice, since FST itself is an additional stressor to mice. In behavior tests, mice were transferred to the experimental room for acclimation at least 1 h prior to testing. All tests took place in a soundproof room between 10:00 and 18:00. After each test, mice were returned to their home cages and then to the holding room.

#### Open field test (OFT)

The OFT was conducted in a quiet room and the open field consisted of an opaque plastic box (30 × 30 × 30 cm). Animals were placed in the center of the open field and allowed to explore for 5 min under dim light. The open field arena was thoroughly cleaned with 70% ethanol between each test. A video tracking system (NeuroVision, Busan, Korea) was used to record the distance traveled as a measure of locomotor activity.

#### Forced swimming test (FST)

The FST was carried out per Porsolt et al.'s protocol^60^. Briefly, mice were placed individually into a glass cylinder (20 cm in height, 14 cm in diameter) filled with 16 cm high water (25 ± 1°C). A divider separated the cylinders so that the mice could not see each other during the trials. After 6 min of the swim test session, immobility time during the final 5-min interval of the test was measured using a video tracking system (EthoVision, Noldus, Wageningen, the Netherlands). Immobility time was defined as the duration a mouse floated passively and made only small movements to keep its nose above the surface.

### Tissue collection and sample preparation for GC-MS analysis

Twenty-four h after the final drug or saline administration, mice were sacrificed by decapitation and their whole brains were removed. The hippocampus was separated from the brain, weighed, rapidly frozen in liquid nitrogen, and stored at −80°C until analysis.

Prior to analysis, 20 mg of mouse hippocampus was extracted in 1 mL of mixture solution of water-methanol-chloroform (2:5:2, v/v/v) with ribitol and alanine-d_4_ as internal standards by homogenization and subsequent centrifugation at 12,300 g for 10 min. Six hundred μL of supernatant was removed and evaporated to dryness in a stream of pure nitrogen gas at room temperature. The dried extract was derivatized into its methoxime derivatives through reaction with 50 μL of 30 mg mL^−1^ methoxyamine hydrochloride solution in pyridine at room temperature for 2 h for protection of ketone groups. Subsequently, trimethylsylation derivatization was performed by reaction with 500 μL of BSTFA at 37°C for 4 h. After derivatization, samples were cooled to room temperature and 1 μL was injected into the gas chromatograph.

### GC-MS analysis

GC-MS analysis was performed using the Hewlett-Packard (HP) GC system 6890 Series equipped with a 5973 Mass Selective Detector (MSD) system. The system was controlled by the Enhanced ChemStation Version B.01.00 program. The GC capillary column was an Agilent J&W HP-5MS UI (30.0 m × 0.25 mm i.d., 0.25 μm film thickness coated 5% diphenyl 95% dimethylpolysiloxane). Helium (purity 99.999%) gas was used as a carrier gas at a constant flow at 1.0 mL min^−1^ and the injection temperature was set at 250°C. Injection was performed in the split mode with the ratio set at 10:1. Column temperature was initially kept at 80°C for 3 min and then increased to 280°C at a rate of 10°C min^−1^, where it was held for 5 min. Temperatures for inlet, ion source, MS quadrupole, and MS transfer line were adjusted at 260°C, 230°C, 150°C, and 280°C, respectively. MS spectra were acquired from *m/z* 45–800.

### Identification of the metabolites

Low molecular weight metabolomes were represented as the chromatographic peaks in total ion current chromatograms (TICs). Peaks with intensity higher than 3-fold of the signal-to-noise (S/N) ratio were recorded and integrated. Identification of these peaks was based on the mass spectra libraries using Wiley 275 L GC-MS Library (Wiley, New York, USA) and some of the peaks were further confirmed using commercially available standards by comparing their MS spectra and retention times.

### Statistical data analysis

Data from the behavioral tests were expressed as means ± standard errors of the mean (SEM). Statistical analysis was carried out using one-way ANOVA test, followed by the student's t-test using Prism 5.0 (GraphPad Software, Inc. USA). Significant differences were indicated at levels of *p* < 0.05, *p* < 0.01, and *p* < 0.001.

Metabolite profiles from the GC-MS analysis were converted into NetCdf file format and subsequently processed by the XCMS online software (https://xcmsonline.scripps.edu) using default settings for peak finding and alignment. The resulting three dimensional matrix containing peak index, sample name, and normalized peak intensity were introduced into SIMCA-P 11.5 software (Umetrics AB, Umea, Sweden), which was used for multivariate statistical analyses including a principal component analysis (PCA), partial least squares-discriminant analysis (PLS-DA), and pair-wise orthogonal projections to latent structures discriminant analyses (OPLS-DA).

## Author Contributions

J.Z., C.G.J. and J.L. designed the experiments. J.Z. and Y.H.J. performed experiments. J.Z. and J.L. wrote the main manuscript text. J.Z., Y.H.J., C.G.J., K.W.C., S.W.K. and J.L. analyzed the data. All authors reviewed the manuscript.

## Supplementary Material

Supplementary Information Supplementary Information

## Figures and Tables

**Table 1:** Summary of the parameters for assessing modeling quality

Groups	No. b	R^2^X_cum_ a	R^2^Y_cum_ a	Q^2^Y_cum_ a
Control-Cms	4	0.841	1.00	0.916
Cms-Flu	3	0.830	0.992	0.942
Cms-Imi	3	0.783	0.990	0.630
Flu-Imi	4	0.870	0.998	0.898

^a^R^2^X_cum_ and R^2^Y_cum_ are the cumulative modeled variation in X and Y matrix, respectively. Q^2^Y_cum_ is the cumulative predicted variation in Y matrix.

^b^Number of components.

**Table 2:** List of differential metabolites for discrimination among Control, Cms, Flu, and Imi groups a

t_R_ (min)	Metabolite	Cms vs. Control			Flu vs. Cms			Imi vs. Cms		
		Fold change	t-test (*p*)	VIP score	Fold change	t-test (*p*)	VIP score	Fold change	t-test (*p*)	VIP score
7.00	Glycine b				1.21	0.021	2.48	1.53	<0.001	3.31
7.36	N-Carboxy-glycine c	0.84	0.021	1.05						
8.57	Valine b							2.11	<0.001	2.07
8.84	Urea b	1.25	0.001	2.43						
9.43	Leucine b				2.08	<0.001	2.2	3.14	<0.001	3.34
9.48	Phosphoric acid c	1.13	0.013	1.51						
9.79	?? d				1.26	0.004	2.21	1.75	<0.001	2.11
12.40	Malic acid b				1.39	<0.001	1.03			
12.82	Asparatic acid b							1.43	<0.001	2.27
13.08	??	0.77	0.017	1.95						
13.28	Creatinine b				0.73	0.006	1.16			
13.99	Glutamic acid b				0.61	<0.001	2	0.61	0.001	4.92
14.60	N-Acetylaspartic acid b				0.64	<0.001	1.9	0.82	0.026	1.31
14.98	Lysine b							2.11	<0.001	1.1
15.08	??	0.58	<0.001	1.47	3.48	<0.001	1.87	2.38	<0.001	1.69
15.73	Glutamine b	1.30	0.008	1.37						
16.06	??				2.16	<0.001	2.79	3.34	<0.001	2.28
18.31	Hexadecanoic acid b	0.81	0.012	2.69	1.54	<0.001	2.46	1.27	<0.001	2.89
19.07	Myo-Inositol b				0.71	0.002	2.23			
19.88	Oleic acid b							1.37	<0.001	1.47
20.09	Octadecanoic acid b	0.85	0.041	2	1.36	<0.001	2.85	1.16	<0.001	3.68
23.50	Adenosine b				0.12	<0.001	3.01	0.04	<0.001	3.35
29.88	Cholesterol b	1.16	0.011	1.16						

^a^Cms, CMS model group; Flu, fluoxetine-treated group; Imi, imipramine-treated group.

^b^Metabolites were identified by using commercially available standards.

^c^Metabolites were identified by comparison with the MS library.

^d^Metabolites were not identified.
