# Supplementary material for: Metabolomic identification of biochemical changes induced by fluoxetine and imipramine in a chronic mild stress mouse model of depression
Source: Sci Rep. 2015 Mar 9;5:8890. doi: 10.1038/srep08890 (PMC4352870; doi:10.1038/srep08890)
Supplement: Supplementary Information [file srep08890-s1.pdf]

Metabolomic identification of biochemical changes induced by fluoxetine and imipramine in a chronic mild stress mouse model of depression

Jing Zhao [a], Yang-Hee Jung [a], Choon-Gon Jang [a], Kwang-Hoon Chun [b], Sung Won Kwon [c], Jeongmi Lee [a, *]

Figure legends

Supplementary Fig. S1. Measurement of body weight at day 0 (a), day 7 (b), day 14 (c), day 21 (d), and day 27 of CMS application. * indicates $p < 0.05$, ** indicates $p < 0.01$, and *** indicates $p < 0.001$. Fluoxetine or imipramine was administered from day 15 of CMS application.

Supplementary Fig. S2. Representative GC-MS chromatograms from the metabolome analysis of control (a), Cms (b), Flu (c), and Imi (d) groups. Peak identification for potential markers: 1, glycine; 2, N-carboxy-glycine; 3, valine; 4, urea; 5, leucine; 6, phosphoric acid; 7, unidentified; 8, malic acid; 9, aspartic acid; 10, unidentified; 11, creatinine; 12, glutamic acid; 13 NAA; 14, lysine; 15, unidentified; 16, glutamine; 17, unidentified; 18, hexadecanoic acid; 19, myo-inositol; 20, oleic acid; 21, octadecanoic acid; 22, adenosine; 23, Cholesterol.

Supplementary Fig. S3. PCA score plot for control and quality control (QC) samples.

Supplementary Fig. S4. S-plots of pair-wise comparison between control and Cms (a), Cms and Flu (b), and Cms and Imi (c). Differential metabolites as potential markers were marked with red squares.

Supplementary Fig. S1.

(a)

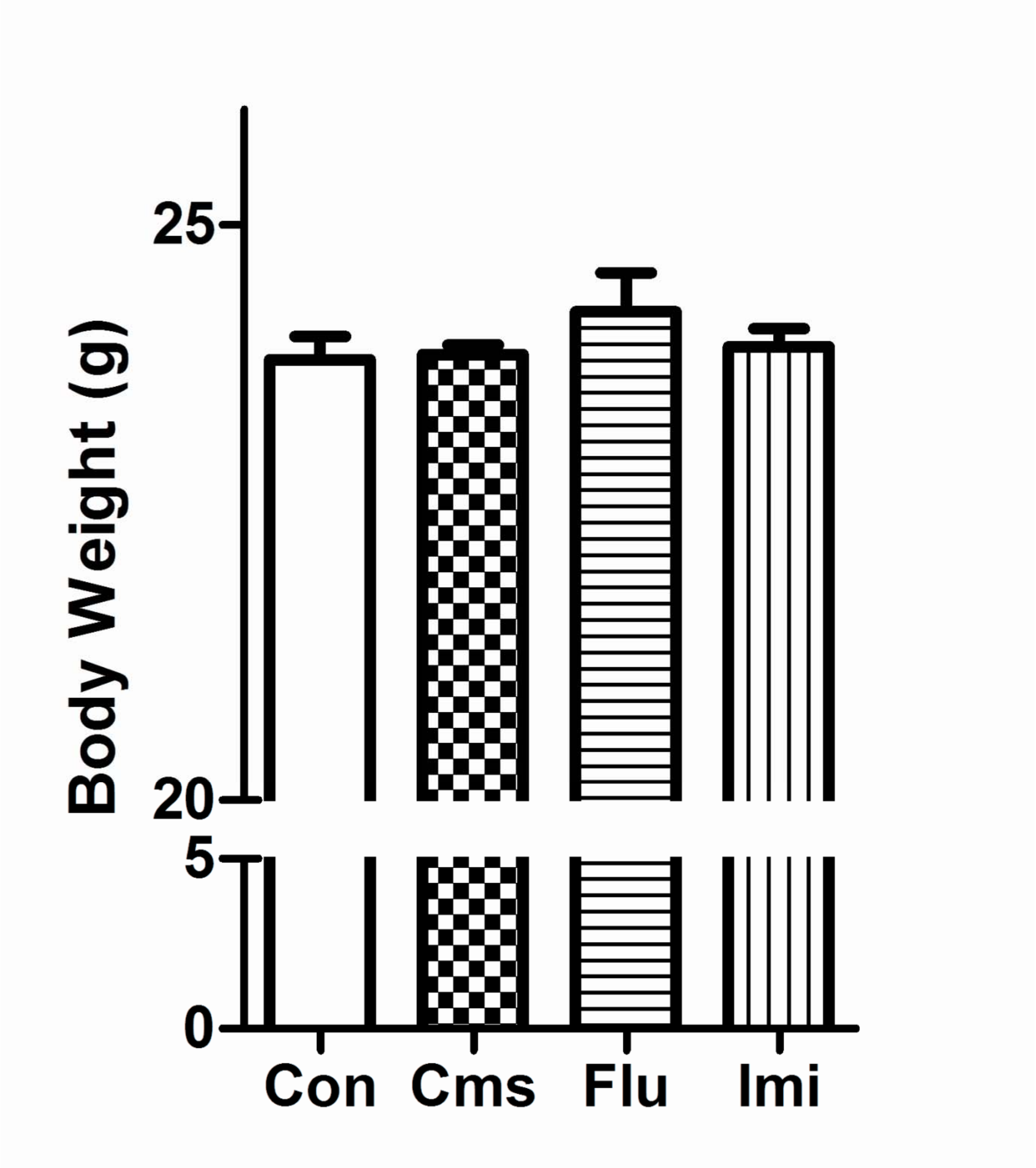

(b)

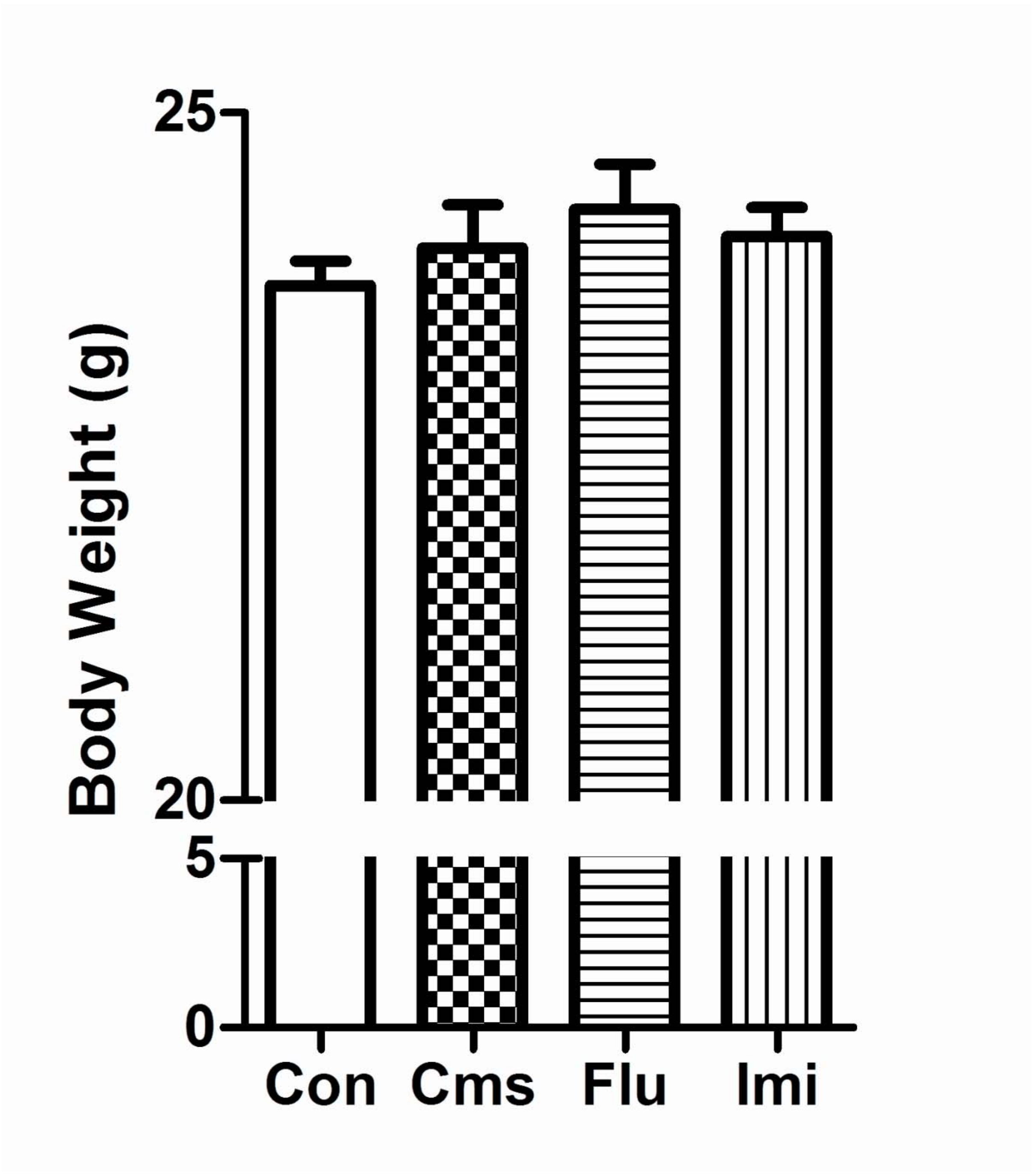

(c)

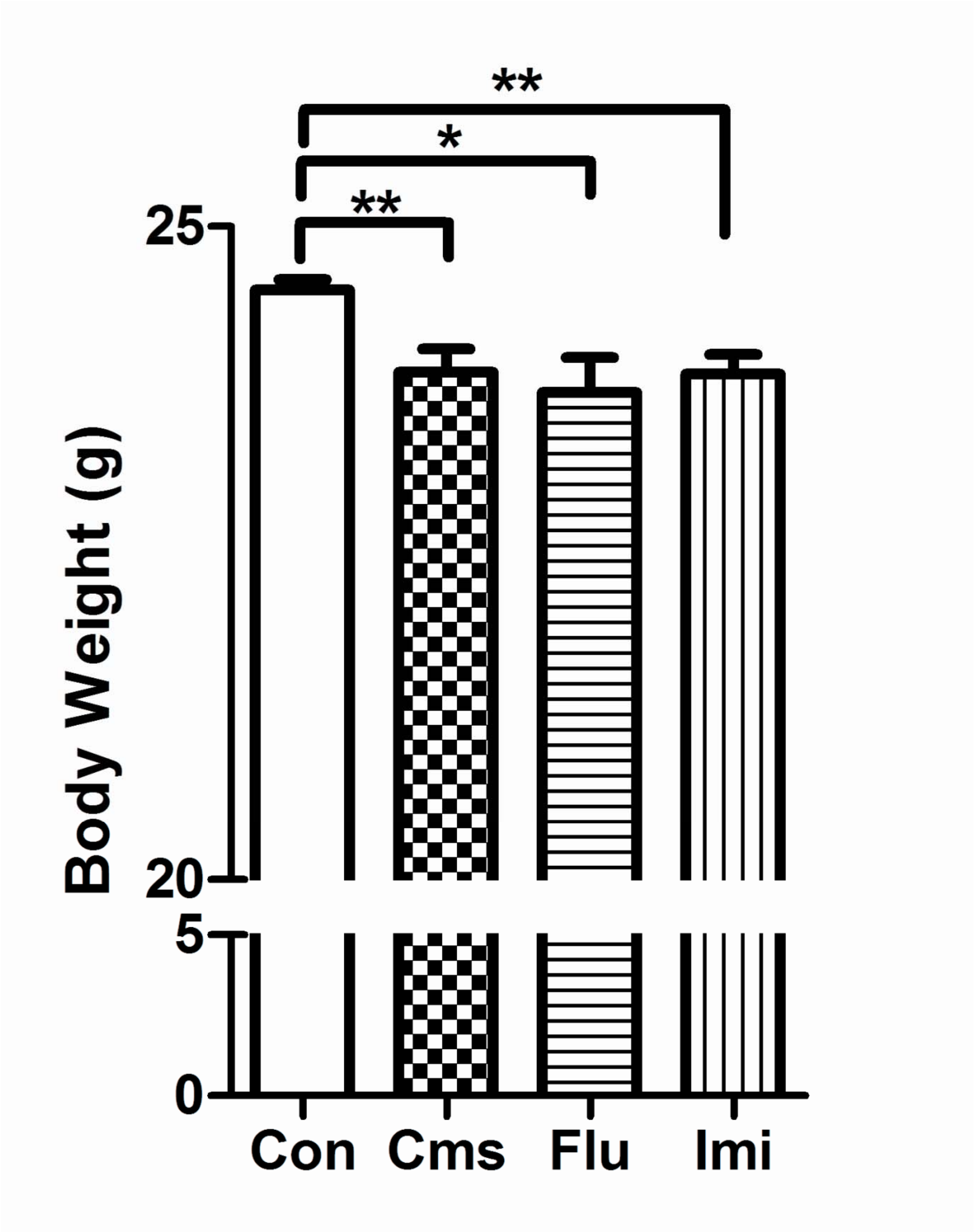

(d)

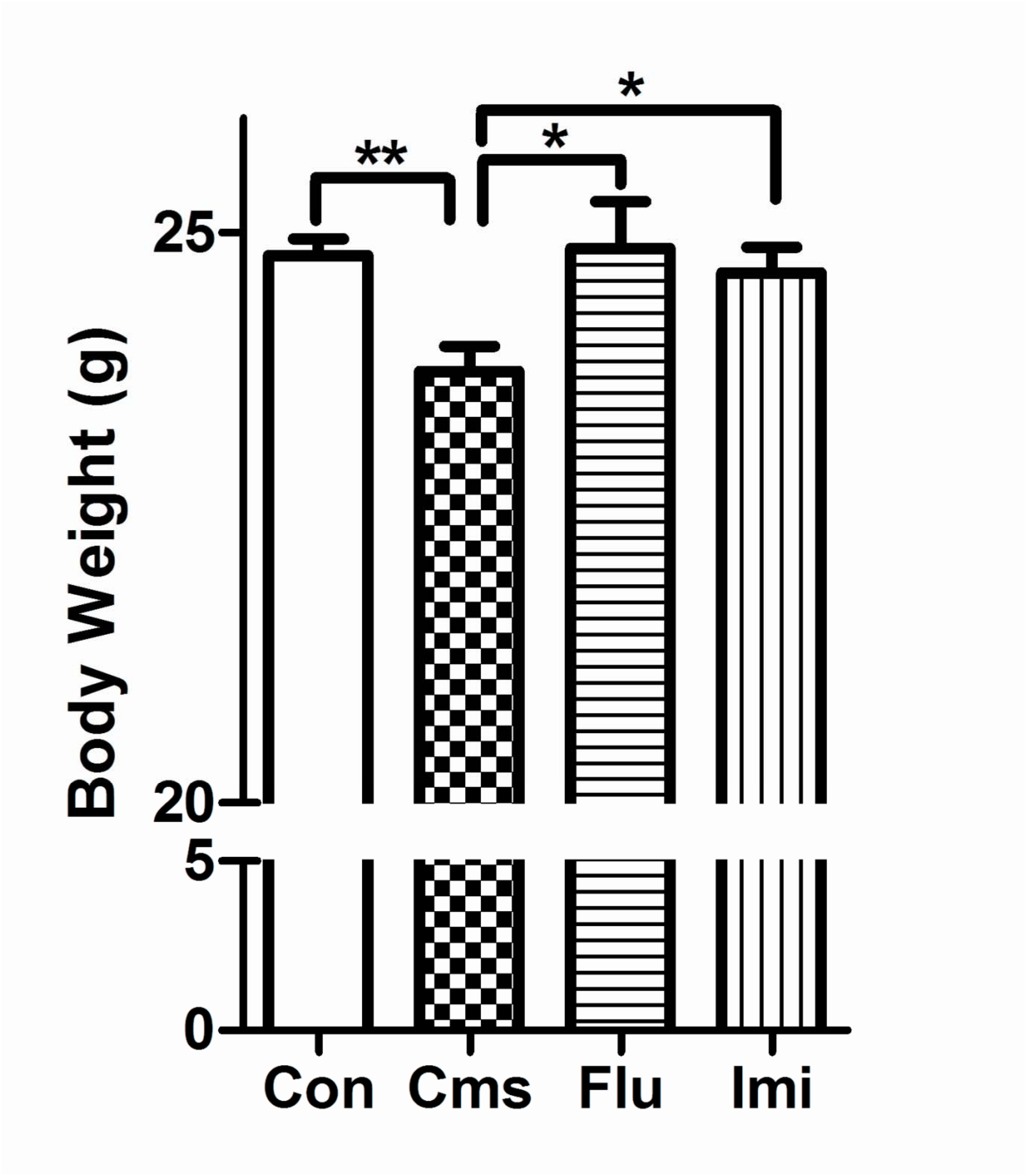

(e)

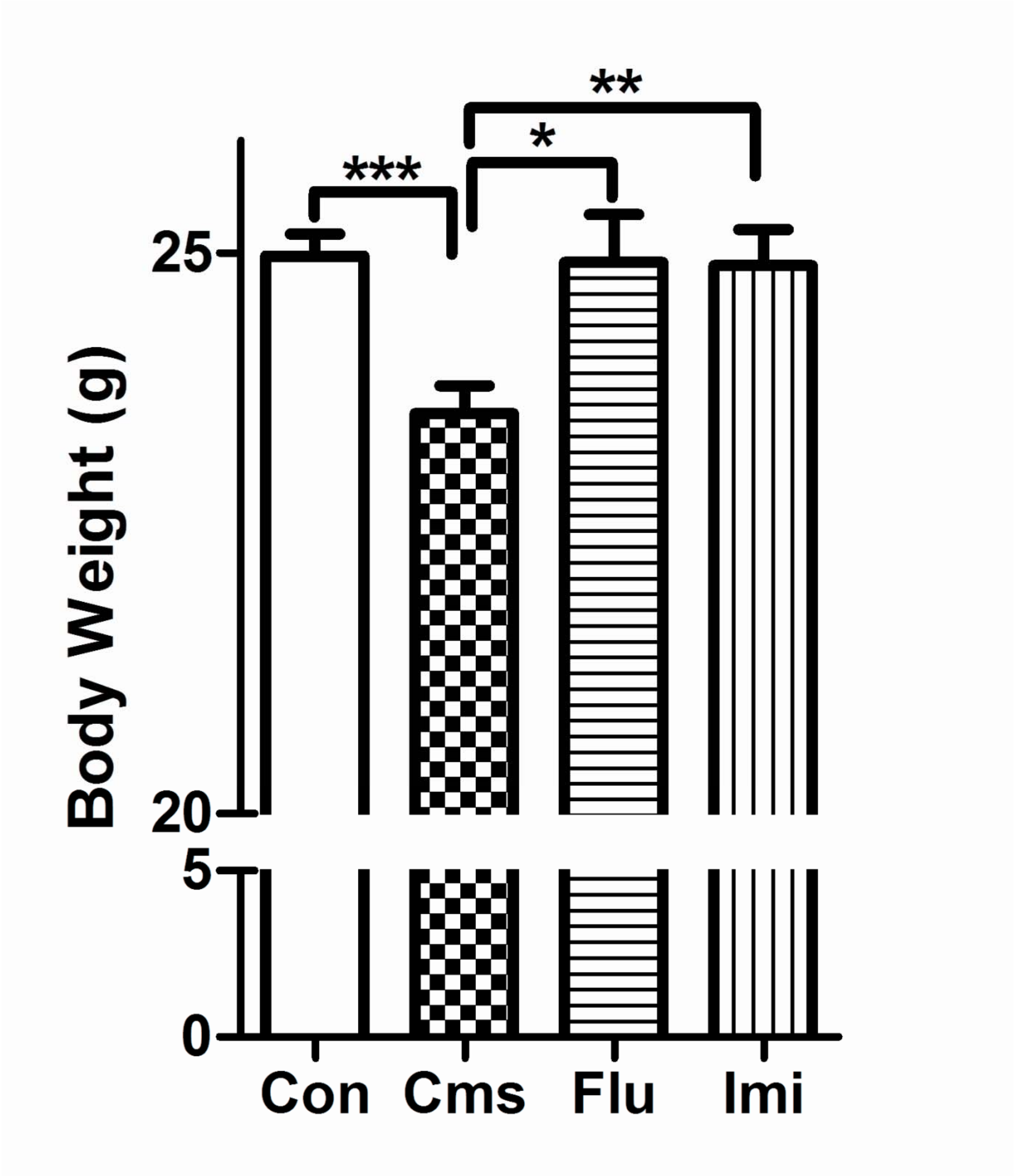

Supplementary Fig. S2.

(a)

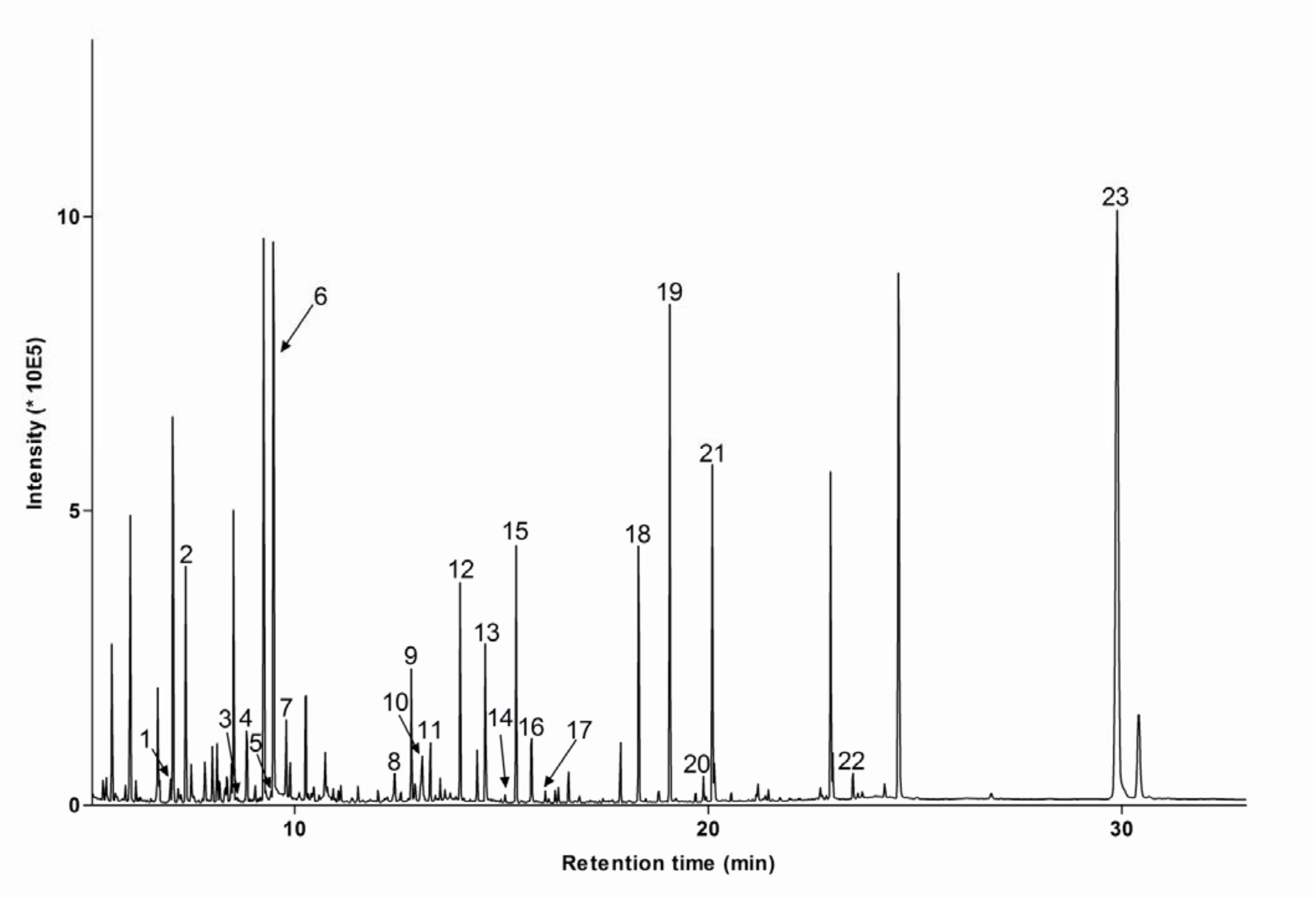

(b)

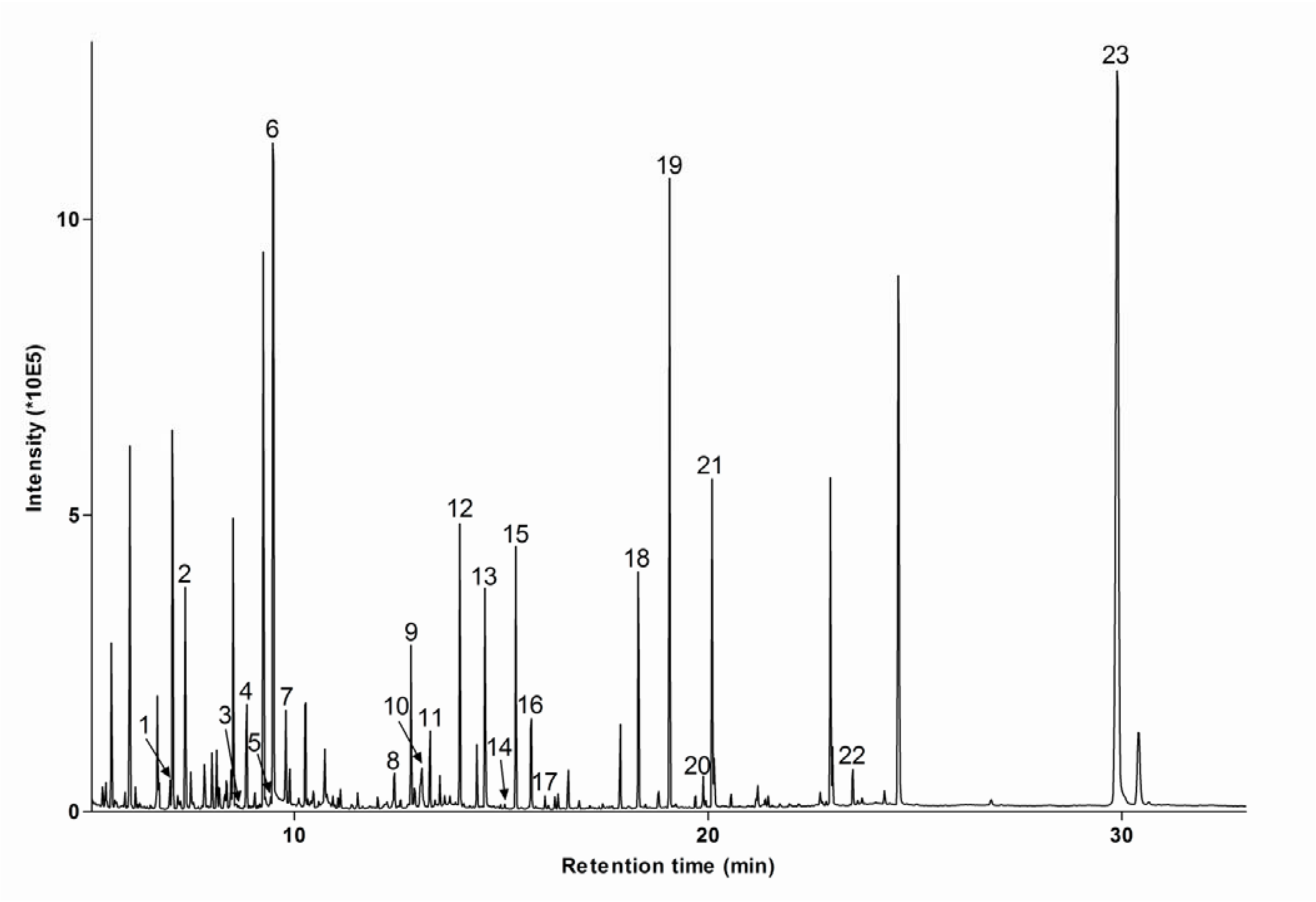

Intensity (*10E5)
0
5
10
Retention time (min)
10
20
30
1
2
3
4
5
6
7
8
9
10
11
12
13
14
15
16
17
18
19
20
21
22
23

(c)

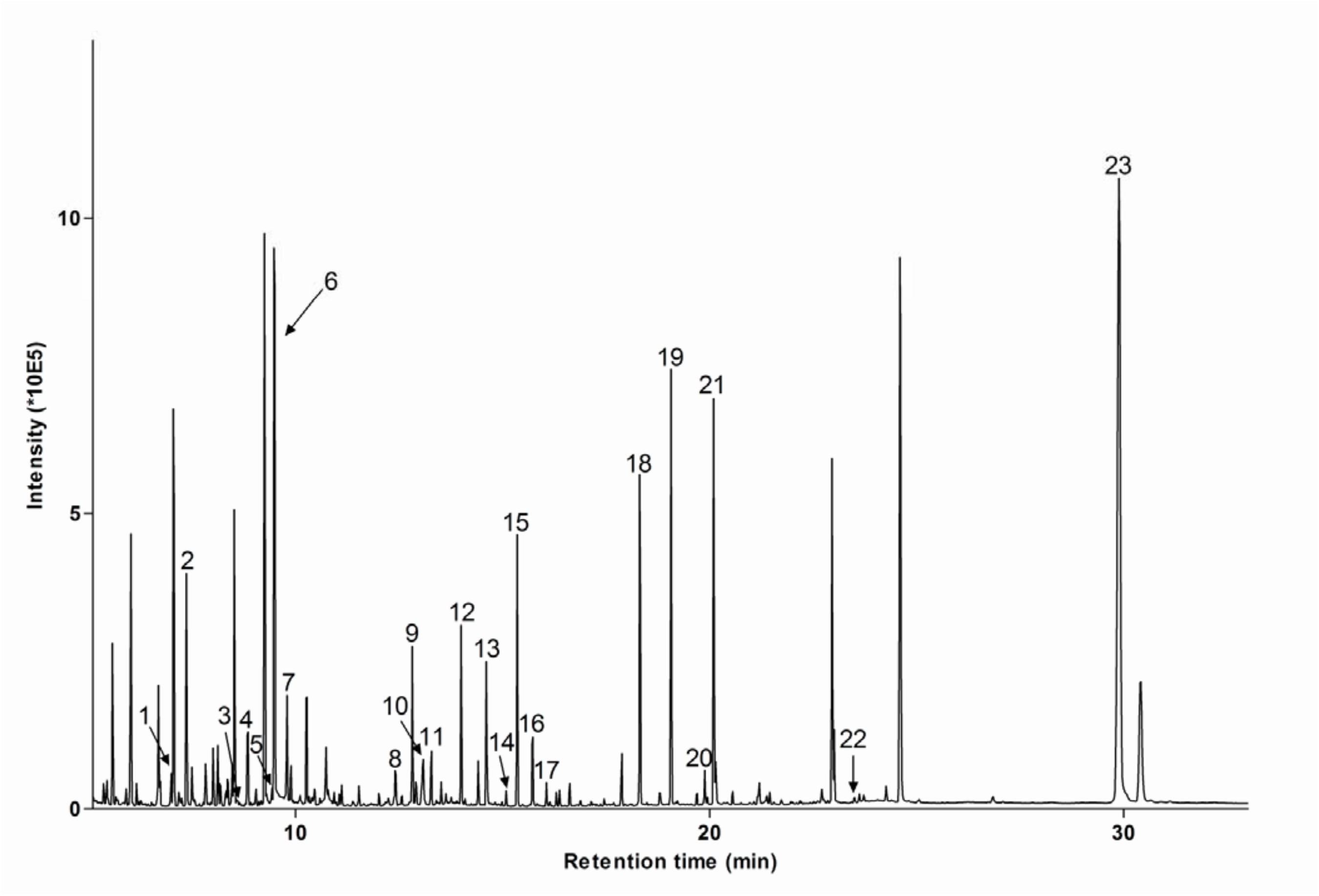

Intensity (*10E5)
Retention time (min)
0
5
10
10
20
30
1
2
3
4
5
6
7
8
9
10
11
12
13
14
15
16
17
18
19
20
21
22
23

(d)

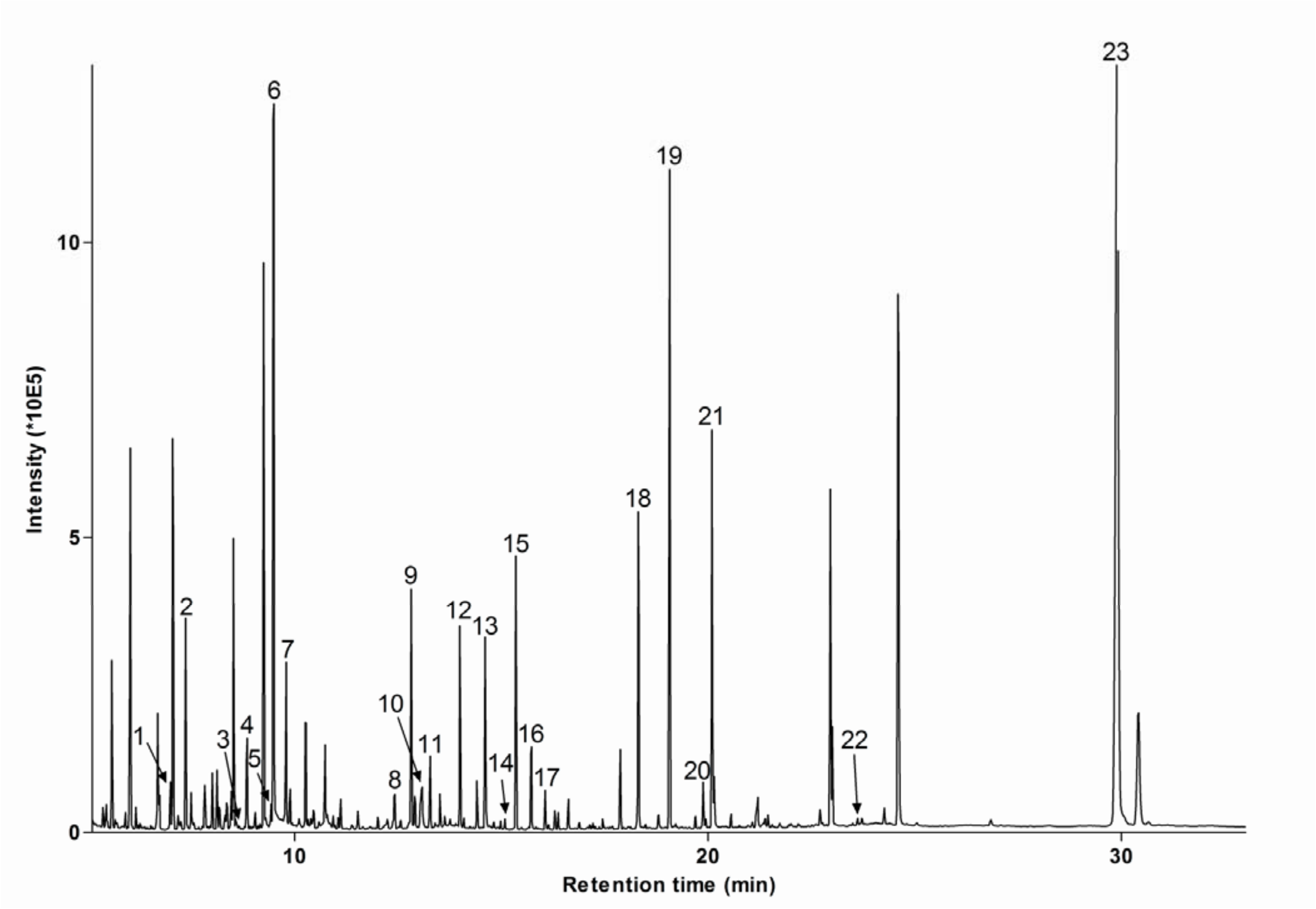

Intensity (*10E5)
Retention time (min)
0
5
10
10
20
30
1
2
3
4
5
6
7
8
9
10
11
12
13
14
15
16
17
18
19
20
21
22
23

Supplementary Fig. S3.

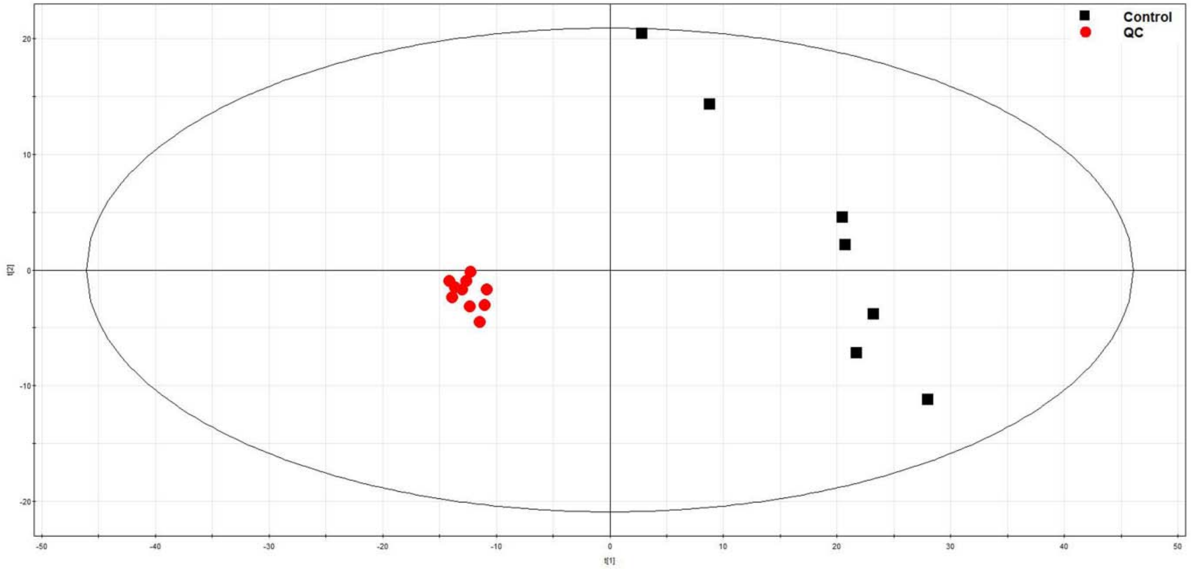

Supplementary Fig. S4.

(a)

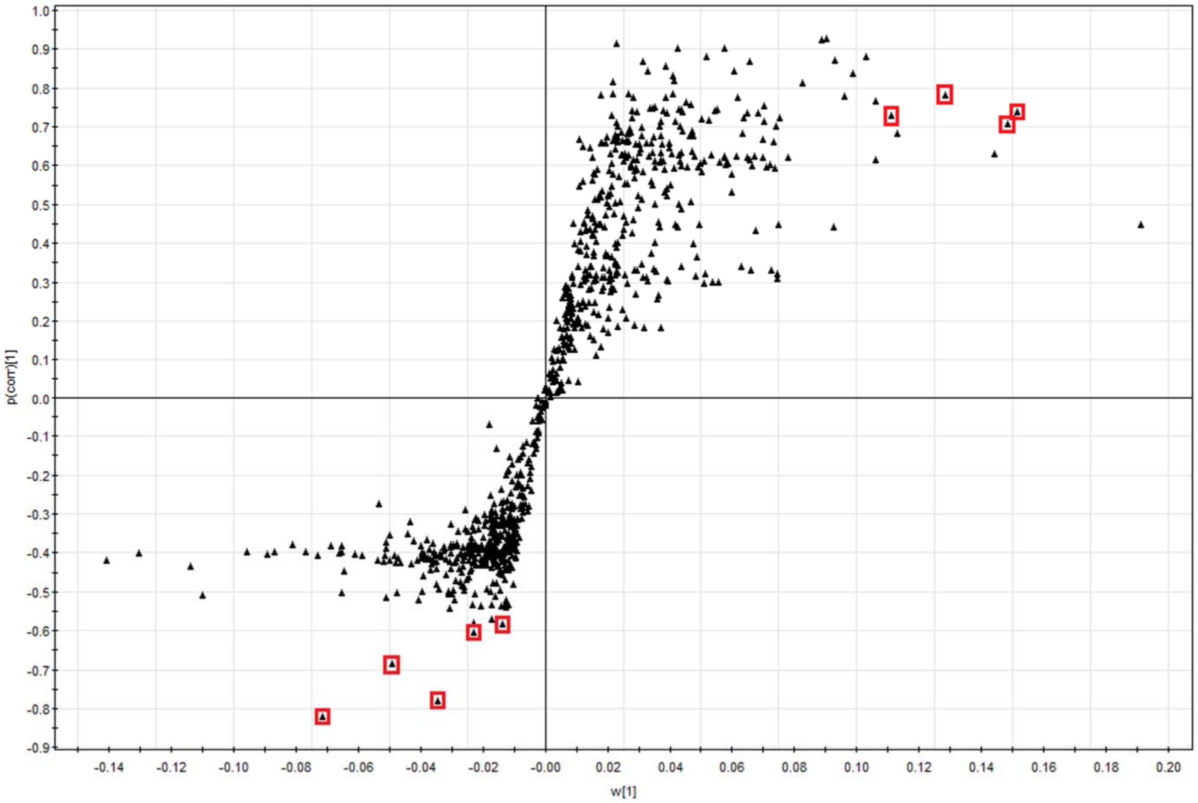

(b)

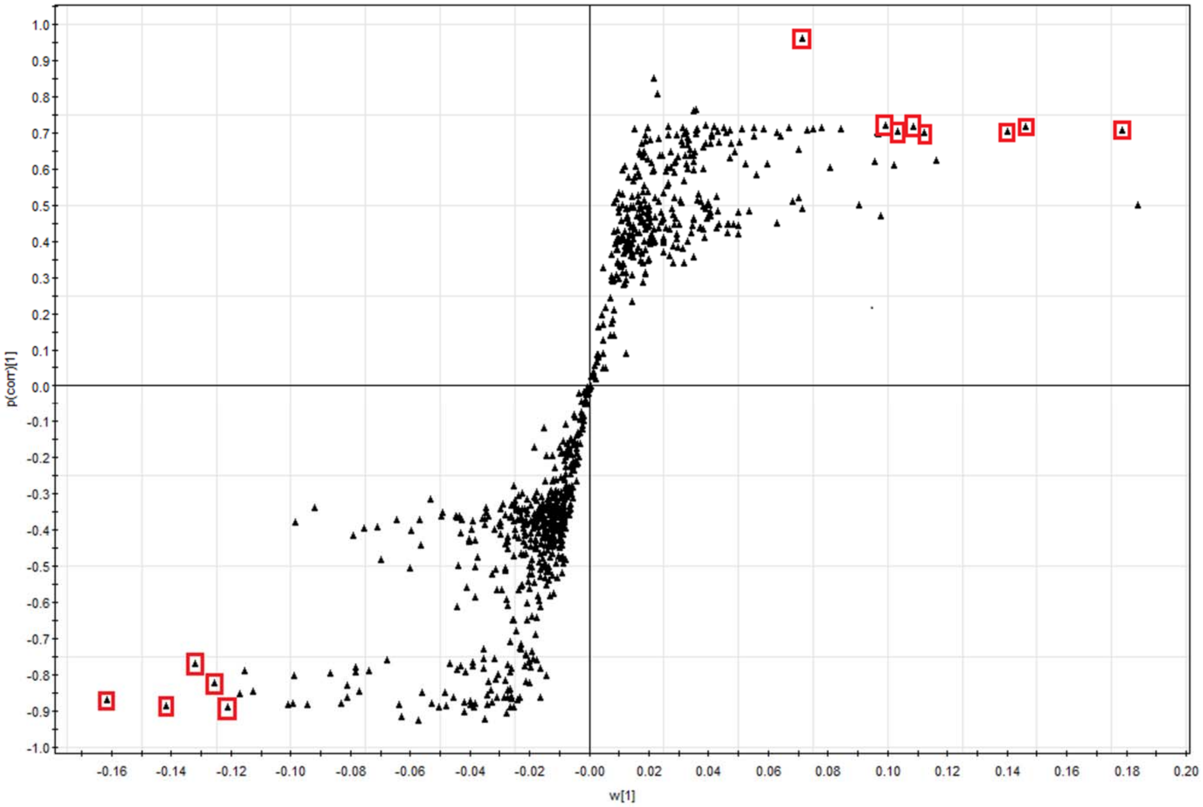
p(corr)[1]
w[1]
1.0
0.9
0.8
0.7
0.6
0.5
0.4
0.3
0.2
0.1
0.0
-0.1
-0.2
-0.3
-0.4
-0.5
-0.6
-0.7
-0.8
-0.9
-1.0
-0.16
-0.14
-0.12
-0.10
-0.08
-0.06
-0.04
-0.02
-0.00
0.02
0.04
0.06
0.08
0.10
0.12
0.14
0.16
0.18
0.20

(c)

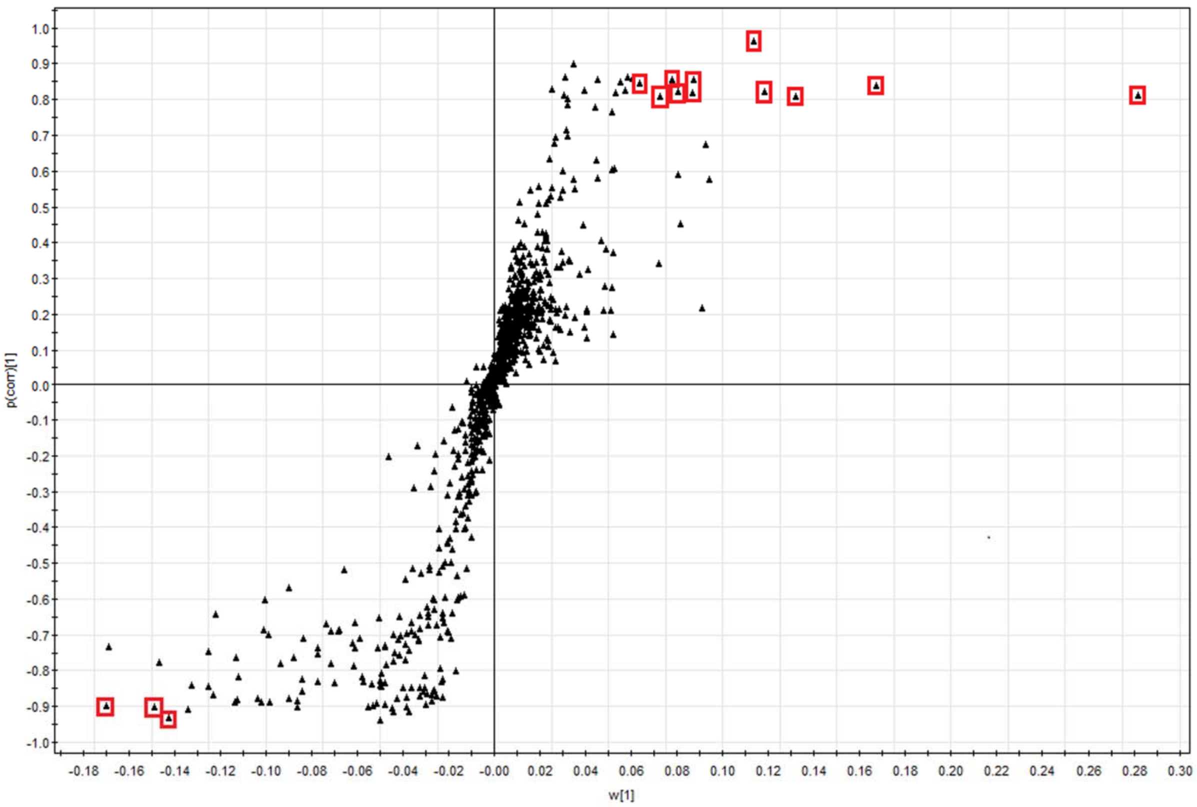

p(corr)[1]
w[1]
1.0
0.9
0.8
0.7
0.6
0.5
0.4
0.3
0.2
0.1
0.0
-0.1
-0.2
-0.3
-0.4
-0.5
-0.6
-0.7
-0.8
-0.9
-1.0
-0.18 -0.16 -0.14 -0.12 -0.10 -0.08 -0.06 -0.04 -0.02 -0.00 0.02 0.04 0.06 0.08 0.10 0.12 0.14 0.16 0.18 0.20 0.22 0.24 0.26 0.28 0.30

Supplementary Table S1. List of identified metabolites in the GC-MS analysis

No.	t_R (min)	Identified metabolites	Mass fragments (*m/z*)	Match factor
1	6.00	Propanoic acid [b]	73, 117, 147, 190	86
2	6.73	Alanine [b]	73, 116, 147	83
3	7.00	Glycine [a]	73, 102, 147, 204	-
4	7.36	N-Carboxy-glycine [b]	73, 133, 147, 200, 235	81
5	8.57	Valine [a]	73, 144, 218	-
6	8.84	Urea [a]	73, 147, 171, 189	-
7	9.43	Leucine [a]	73, 147, 158	-
8	9.48	Phosphoric acid [a]	73, 299, 314	-
9	10.73	L-Lactic acid [b]	73, 147, 204, 218	80
10	11.11	L-Threonine [a]	73, 117, 147, 218, 291	-
11	12.40	Malic acid [a]	73, 147, 233, 335	-
12	12.42	Butanedioic acid [b]	73, 147, 233, 245	81
13	12.82	Aspartic acid [a]	73, 100, 147, 218, 232	-
14	13.28	Creatinine [a]	73, 115, 147, 256, 314	-
15	13.99	Glutamic acid [a]	73, 128, 147, 156, 246	-
16	14.09	L-Phenylalanine [a]	73, 100, 115, 147, 192, 218	-
17	14.24	Dodecanoic acid [a]	73, 117, 129, 145, 257	-
18	14. 60	N-Acetylaspartic acid [a]	73, 147, 274, 326, 376	-
19	14.98	Lysine [a]	73, 84, 147, 156, 299, 362	-
20	15.73	Glutamine [a]	73,147, 155, 245, 299, 357	-

21	15.83	1-Naphthalenecarboxylic acid [b]	57, 127, 151, 185, 229, 244	80
22	16.29	Citric acid [a]	73, 147, 273, 347, 363, 375	-
23	16.37	Tetradecanoic acid [b]	73, 117, 131, 145, 285	98
24	17.46	L-Tyrosine [b]	73, 147, 218, 280	89
25	17.88	L-Altrose [b]	73, 147, 217, 305, 319	82
26	18.31	Hexadecanoic acid [a]	55, 73, 117, 132, 145, 313	-
27	18.80	Heneicosane [b]	57, 71, 85, 99, 299	98
28	19.07	Myo-Inositol [a]	73, 147, 191, 204, 217, 265, 305, 318	-
29	19.22	Heptadecanoic acid [b]	73, 117, 145, 299, 327, 342	83
30	19.70	Docosane [b]	57, 71, 85, 99, 310	99
31	19.88	Oleic acid [a]	73, 117, 129, 145, 264, 339	-
32	20.09	Octadecanoic acid [a]	73, 117, 132, 145, 341	-
33	20.56	Ticosane [b]	57, 71, 85, 281, 324	95
34	21.39	Eicosane [b]	57, 71, 85, 99, 281	98
35	21.95	Myo-Inositol-2-phosphate [b]	73, 147, 217, 299, 318, 387	88
36	22.22	Pyrimidine [b]	73, 147, 265, 318, 387	86
37	23.50	Adenosine [a]	73, 217, 230, 245, 340	-
38	29.88	Cholesterol [a]	129, 329, 353, 368, 458	-

[a] Metabolites were identified by using commercially available standards.

[b] Metabolites were identified by comparison with the MS library.

Supplementary Table S2. List of differential metabolites between experimental group (Cms, Flu, Imi)[a] and control groups.

t_R (min)	Metabolite	Cms vs. Con			Flu vs. Con			Imi vs. Con		
		Fold Change	t-test (*p*)	VIP score	Fold Change	t-test (*p*)	VIP score	Fold Change	t-test (*p*)	VIP score
7.00	Glycine [b]							1.77	<0.001	2.16
7.36	N-Carboxyglycine [c]	0.84	0.021	1.05						
8.57	Valine [b]				1.69	<0.001	1.55	1.47	<0.001	1.44
8.84	Urea [b]	1.25	0.001	2.43						
9.43	Leucine [b]				2.28	<0.001	2.40	3.13	<0.001	2.41
9.48	Phosphoric acid [b]	1.13	0.013	1.51				1.20	0.001	2.14
9.79	?? [d]				1.54	<0.001	2.15	2.29	<0.001	2.20
12.82	Aspartic acid [b]							1.68	<0.001	1.61
13.08	??	0.77	0.017	1.95						
15.08	??	0.58	<0.001	1.47	3.28	<0.001	1.25			
15.73	Glutamine [b]	1.30	0.008	1.37						
16.06	??				1.87	<0.001	2.46	2.86	<0.001	2.44
18.31	Hexadecanoic acid [b]	0.81	0.012	2.69						
20.09	Octadecanoic acid [b]	0.85	0.041	2.00						
23.50	Adenosine [b]							0.06	<0.001	2.38
29.88	Cholesterol [b]	1.16	0.011	1.16				1.26	<0.001	1.34

[a] Cms, CMS model group; Flu, fluoxetine-treated group; Imi, imipramine-treated group.

[b] Metabolites were identified by using commercially available standards.

[c] Metabolites were identified by comparison with the MS library.

[d] Metabolites were not identified.
